# Cellular Aspects of Muscle Specialization Demonstrate Genotype – Phenotype Interaction Effects in Athletes

**DOI:** 10.3389/fphys.2019.00526

**Published:** 2019-05-08

**Authors:** Martin Flück, Manuel Kramer, Daniel P. Fitze, Stephanie Kasper, Martino V. Franchi, Paola Valdivieso

**Affiliations:** Laboratory for Muscle Plasticity, Department of Orthopedics, Balgrist University Hospital, University of Zürich, Zurich, Switzerland

**Keywords:** muscle, athlete, myofibril, mitochondria, capillary, gene, angiotensin, tenascin

## Abstract

**Introduction:**

Gene polymorphisms are associated with athletic phenotypes relying on maximal or continued power production and affect the specialization of skeletal muscle composition with endurance or strength training of untrained subjects. We tested whether prominent polymorphisms in genes for angiotensin converting enzyme (ACE), tenascin-C (TNC), and actinin-3 (ACTN3) are associated with the differentiation of cellular hallmarks of muscle metabolism and contraction in high level athletes.

**Methods:**

Muscle biopsies were collected from *m. vastus lateralis* of three distinct phenotypes; endurance athletes (*n* = 29), power athletes (*n* = 17), and untrained non-athletes (*n* = 63). Metabolism-, and contraction-related cellular parameters (such as capillary-to-fiber ratio, capillary length density, volume densities of mitochondria and intramyocellular lipid, fiber mean cross sectional area (MCSA) and volume densities of myofibrils) and the volume densities of sarcoplasma were analyzed by quantitative electron microscopy of the biopsies. Gene polymorphisms of ACE (I/D (insertion/deletion), rs1799752), TNC (A/T, rs2104772), and ACTN3 (C/T, rs1815739) were determined using high-resolution melting polymerase chain reaction (HRM-PCR). Genotype distribution was assessed using Chi^2^ tests. Genotype and phenotype effects were analyzed by univariate or multivariate analysis of variance and *post hoc* test of Fisher. *P*-values below 0.05 were considered statistically significant.

**Results:**

The athletes demonstrated the specialization of metabolism- and contraction-related cellular parameters. Differences in cellular parameters could be identified for genotypes rs1799752 and rs2104772, and localized *post hoc* when taking the interaction with the phenotype into account. Between endurance and power athletes these concerned effects on capillary length density for rs1799752 and rs2104772, fiber type distribution and volume densities of myofibrils (rs1799752), and MSCA (rs2104772). Endurance athletes carrying the I-allele of rs1799752 demonstrated 50%-higher volume densities of mitochondria and sarcoplasma, when power athletes that carried only the D-allele showed the highest fiber MCSAs and a lower percentage of slow type muscle fibers.

**Discussion:**

ACE and tenascin-C gene polymorphisms are associated with differences in cellular aspects of muscle metabolism and contraction in specifically-trained high level athletes. Quantitative differences in muscle fiber type distribution and composition, and capillarization in knee extensor muscle explain, in part, identified associations of the insertion/deletion genotypes of ACE (rs1799752) with endurance- and power-type Sports.

## Introduction

Skeletal muscle is a critical determinant of Sports performance. This influence has its origin in the force producing capacity of contracting muscle which is fueled by the conversion of metabolic substrates. These relationships are specifically evident for muscle output at the extremes of the power spectrum, which characterize the capacity for producing maximal power and the capacity to sustain power output (i.e., fatigue resistance) (Flueck, 2010). In a healthy, able-bodied and motivated subject (Hegner, 2012; Speckmann et al., 2015), both capacities are to a considerable degree set by the structural characteristics of skeletal muscle: on one side maximal power and force is related to cross-sectional area and the volume density of myofibrils of muscle fiber (types), and on the other fatigue resistance (or endurance) is related to the capillarization, and the volume densities of mitochondria and intramycellular lipid in muscle fibers (Vock et al., 1996; Weibel, 1996).

The performance of skeletal muscles can be specifically developed (or maximized) by physical training-through a feed forward mechanism that remodels muscle composition. In this respect, maximal performance is usually improved with a high-load, low-repetitive type of training whereas fatigue resistance is ameliorated to a considerable extent by a highly-repetitive low-load type of training (Wernbom et al., 2007; Flueck and Eilers, 2010). The respective functional improvement can be explained in terms of quantitative and qualitative structural adaptations at the level of muscle organelles that set contractile function and metabolic supply. Accordingly, an improvement in maximal power can represent an increase in the cross-sectional area of muscle fiber (types) and increased volume density of myofibrils (Speckmann et al., 2015). On the contrary, an increase in fatigue resistance can be explained by increases in capillary-to-fiber ratio or capillary length density in locomotor muscle, in conjunction with an elevated volume density of mitochondria and intramyocellular lipid (Oberholzer et al., 1976; Ingjer, 1979; Speckmann et al., 2015).

There is classical evidence that muscle composition and muscle performance (i.e., peak force and endurance) and systemic components of endurance performance (i.e., maximal oxygen uptake, VO_2_max) are influenced by heritable factors (Klissouras et al., 1973; Simoneau and Bouchard, 1995; Bouchard et al., 1999; Silventoinen et al., 2008). Natural sequence variants in genes, i.e., gene polymorphisms, have been identified to be associated with specific athletic traits, i.e., power and endurance (Yang et al., 2003; Bray et al., 2009; Aleksandra et al., 2016). Polymorphisms in the genes for actinin-3 (ACTN3; rs1815739), myostatin (rs1805086 and rs1805065), angiotensin converting enzyme (ACE; rs1799752), and tenascin-C (TNC; rs2104772) have been found to be associated with differences in muscle composition and/or muscle volume in untrained and moderately trained individuals and adaptations in muscle composition with endurance training (Vincent et al., 2007; Santiago et al., 2011; Vaughan et al., 2013; Li et al., 2014; Vaughan et al., 2016; Valdivieso et al., 2017a). Specifically the I-allele represented by polymorphism rs1799752, which characterizes the insertion of a silencer region within intron 16 of the ACE gene, is found to be associated with superior trainability of endurance performance compared to subject being characterized by the homozygous deletion of the silencer region (i.e., ACE-D/D genotypes; (Woods et al., 2000; Flueck et al., 2010). ACE I-allele carriers (i.e., ACE-I/I and ACE-I/D genotypes) have been found to demonstrate lower ACE transcript expression in skeletal muscle, and reduced serum levels of the encoded ACE, and its product, the major vasoconstrictor angiotensin 2, than ACE-D/D genotypes (Rigat et al., 1990; Vaughan et al., 2013; Mathes et al., 2015; Vaughan et al., 2016). Consequently ACE-I/I and ACE-I/D genotypes show increased capillary perfusion during exhaustive endurance exercise when capillary perfusion is not improved in ACE-D/D genotypes (van Ginkel et al., 2015). The reportedly reduced improvement of blood supply in ACE-D/D compared to ACE-I/I and I/D genotypes is related to an inefficient import of serum glucose, perturbed mitochondrial metabolism (Vaughan et al., 2016), and lower transcript expression of lipid and glucose metabolism-associated factors in knee extensor muscle of ACE-D/D genotypes at the end of exhaustive endurance exercise (Mathes et al., 2015). Consistently, ACE I-allele carriers are found to demonstrate greater increases in the volume density of subsarcolemmal mitochondria, and intramyocellular lipid stores in *m. vastus lateralis* after endurance training (Vaughan et al., 2013). The relevance of the ACE-related response of *m. vastus lateralis* to endurance exercise, and the contribution of its genetic inhibition via the ACE I-allele, is corroborated by the effects of pharmacological ACE inhibition on metabolism-related transcript in *m. vastus lateralis* post endurance exercise and training (Zoll et al., 2006; van Ginkel et al., 2016), and the fact that this shift in transcript expression is modulated by the ACE-I/D gene polymorphism (Mathes et al., 2015). For instance, oral intake of the ACE inhibitor lisinopril increased transcript levels of the shear stress-related pro-angiogenic factors VEGF and tenascin-C in *m. vastus lateralis* when the levels of hypoxia-related mitochondrial transcripts were lowered (van Ginkel et al., 2015). Additionally, ACE I-allele carriers are found to demonstrate a higher cross-sectional area of *m. vastus lateralis* and embedded muscle fibers compared to endurance-trained ACE-D/D genotypes (Vaughan et al., 2016; Valdivieso et al., 2017b). Similarly, polymorphism rs2104772 being characterized by the non-synonymous exchange of thymidine (T)-to-adenosine (A) in amino acid codon 1677 of tenascin-C has been found associated with higher volume densities of mitochondria and higher gains in capillary-to-fiber ratio in *m. vastus lateralis* with endurance exercise (Valdivieso et al., 2017a). These differences were related to lowered muscle levels of tenascin-C protein in T/T homozygotes respective to A-nucleotide carriers, reproducing the negative effects of a lowered tenascin-C expression on activity-induced angiogenesis as seen in anti-gravity muscles of tenascin-C deficiency transgenic mice (Fluck et al., 2008). As well, ACE I-allele carriers (i.e., polymorphism rs1799752; Zhang et al., 2003; Fluck et al., 2008) and T/T-genotypes of polymorphism rs1805086 in the ACTN3 gene (Vincent et al., 2007), being characterized by the absence of ACTN3 protein, have been found to demonstrate a higher percentage of slow type muscle fibers in healthy untrained subjects, and this corroborates with observations in the respective transgenic deficient animals (Zhang et al., 2005; MacArthur et al., 2008).

Endurance exercise during months of training has been identified to modify certain genotypic influences on muscle composition. For instance, in healthy subjects endurance training has been found to affect – and in part overrule – the influence of the ACE-I/D genotype on the concentration of metabolic substrates and transcript expression (Valdivieso et al., 2017b). As this involves certain signaling processes of muscle plasticity, i.e., the pro-angiogenic factor VEGF (discussed in Valdivieso et al., 2017b), genetic influences being reported in untrained individuals may not linearly translate to well- and highly-trained subjects. This may specifically apply to the adaptive mechanisms in high level athletes, whose muscles are subjected to the impact of high loads and intensities during years of training and competition. This influence may outweigh the influence of a single genetic factor (discussed in Frese et al., 2016; Leonska-Duniec et al., 2016).

It has not been investigated whether differences in muscular performance in an elite athlete population are related to genetic influences on the composition of muscle fibers. Taking into account the immediate relevance of muscle fiber composition for muscle performance (Hoppeler, 1986; Weibel, 1996; Vock et al., 1996; Flück and Hoppeler, 2003; Zierath and Hawley, 2004; Harber and Trappe, 2008), adding insights into the interrelationships between fitness-associated gene polymorphisms and muscle composition would be an important complement to understand individual responses to training. It may allow developing a future personalized approach to physical training which takes the trainability of metabolic and contractile traits of muscle performance into account. The aim of our investigation was therefore to test whether differences in cellular aspects of muscle metabolism and contraction in high level endurance- and power-athletes are associated with either of the three genotypes (rs1799752, rs2104772, rs1815739), as this has in part been shown for untrained or moderately-trained subjects, and transgenic animals, and to assess to what extent this depends on the athletic phenotype. We chose to investigate the knee extensor muscle, *m. vastus lateralis*, due to its involvement in propulsion and established biologically relevant linear relationships between its composition and systemic/physiological measures of exercise performance (Tihanyi et al., 1982; Valdivieso et al., 2017a; Pollock et al., 2018; van der Zwaard et al., 2018). Based on previous findings in healthy subjects we expected that the ACE-I/D and TNC gene polymorphisms, respectively rs1799752 and rs2104772, would affect (aerobic) metabolism- and contraction-related structural aspects of muscle, which are conditioned by endurance training. Conversely, we expect that the ACTN3 genotype (rs1815739) would affect the cellular composition of muscle fibers related to contractile function, assuming that this would be more evident in power athletes.

## Materials and Methods

### Study Design

The present study was carried out in the Laboratory for Muscle Plasticity at the research center Balgrist Campus of the Balgrist University Hospital. Frozen biopsies from the *m. vastus lateralis* muscle of high level competitive athletes from endurance- and power-type Sports were collected during the athletes’ active phase, and healthy untrained individuals (i.e., non-athletes) were genotyped according to the polymorphisms for the insertion/deletion allele of ACE gene (ACE-I/D, rs1799752) and single nucleotide polymorphisms in the genes for ACTN3 (C/T rs1815739) and TNC (A/T, rs2104772). The samples stem from various published and unpublished studies for which data on muscle composition, age, gender, and aerobic characteristics of performance, and the level of athletic specialization were available (Supplemental Table S1; Hoppeler, 1986; Luthi et al., 1986; Hochli et al., 1995; Suter et al., 1995; Billeter et al., 2003). Additionally, fiber typing has been performed for the purpose of this investigation on frozen material.

### Ethics Statement

The study was conducted in accordance with the Helsinki declaration for research on humans. The gathering of physiological and cell biological meta-data was conducted with permission of the Ethics committee of the canton of Berne. Genotyping was carried out and analyzed anonymously under application of the non-competence rule of the Human Research Act after clarification by the ethics committee of the canton of Zurich.

### Subjects

The sample consisted of 30 elite white Caucasian athletes from endurance-type (professional cyclists, 100 km runners) and 17 white Caucasian power- type (shot-putters, weight lifters) Sports that lived in Switzerland. The athletes included participants and winners of international competitions, world championships and Olympic Games; and ranked at the top of their respective disciplines. The untrained individuals (i.e., non-athletes) were recruited previously from employees and students being associated with the Universities of Berne or Fribourg. Further prerequisites for inclusion in the study were the presence of sufficient biopsy material for genotyping and well-documented data on skeletal muscle composition. Table 1 shows the subject characteristics.

**Table 1:** Characteristics of the three phenotypes.

Parameter	Age (years)	Body mass (kg)
Endurance athlete (*n* = 30)	30.1 ± 5.7	64.5 ± 6.0
Power athlete (*n* = 17)	25.4 ± 10.1	88.6 ± 25.2
Non-athlete (*n* = 63)	29.5 ± 9.3	76.5 ± 12.8
*p*-value (3 phenotypes)	0.147	0.001
*p*-value (Endurance vs. Power)	0.455	< 0.001


*Mean ± standard deviation of age and body mass of the studied endurance and power athletes, and non-athletes. Statistical analysis was conducted with univariate ANOVA with post hoc test of least significant difference.*

### Muscle Composition

Biopsies were characterized for muscle ultrastructure with an established morphometric technique based on electron micrography of glutaraldehyde-fixed muscle biopsies (Schmutz et al., 2006). The following parameters of the athletes were analyzed and have in part been reported (Hoppeler, 1986; Hochli et al., 1995; Billeter et al., 2003): Capillary-to-fiber ratio, capillary length density, mean fiber cross sectional area, myofibrillar volume density, mitochondrial volume density, sarcoplasmic volume density, intramyocellular lipid density. Sarcoplasmic volume density was defined as structures internal to muscle fibers which did not represent myofibrils, mitochondria and intramyocellular lipid; therefore representing the sum of the membranous structures (sarcoplasmic reticulum, Golgi-apparatus), cytosol and myonuclei (Horber et al., 1987; Vock et al., 1996; Weibel, 1996). The distribution of slow and fast type muscle fiber types was determined based on histochemical ATPase staining or immunofluorescence as described (Supplemental Figure S1; Billeter et al., 2003; Fluck et al., 2019). The fast fiber population was not differentiated into the different subtypes. The comparison of values for the histochemical and immunological detection of slow and fast fiber types in consecutive sections revealed a mean error or measurement of less than 1%.

### DNA Extraction and Genotyping

For DNA extraction and genotyping, the collected muscle tissue from the *m. vastus lateralis* was used. If for some reason the biopsy from the *m. vastus lateralis* was not usable, samples from the *m. deltoideus* were used. All muscle biopsies were stored at minus 80°C. In order to collect 5 mg of muscle tissue, 25 μm cryosections were taken at -25°C. If it was not possible to collect 5 mg of tissue in certain biopsies, an attempt was made to continue with the available amount of muscle tissue in the next working steps. Experience shows that reliable genotyping can also be carried out with a smaller amount of muscle tissue. The samples were then stored at minus 80°C until the DNA extraction step. The DNA was extracted using DNeasy Blood & Tissue Kit (Cat. No 69504, Qiagen, Basel, Switzerland). In order to extract a high quantity of DNA for genotyping, several lysis, and washing steps followed by filtration by centrifugation were carried out.

For the high HRM-PCR, an identical procedure was used for all three investigated genotypes. The reaction mix was prepared with the KAPA HRM FAST Master Mix (KAPA BIOSYSTEMS, Labgene Scientific, Châtel-St-Denis, Switzerland) and specifically designed primers.

For detecting TNC SNP rs210477, two primers (5′-CAAAAAAAGCAGTCTCTGAGCCAC-3′ and 5′-TTCAGTAGTCTCTCTCTGAGAC-3′) were used as established (Valdivieso et al., 2017a) to amplify a 85 base pair long DNA fragment. For detecting ACTN3 SNP rs1815739, the primers ACTN3 forward (5′-CTGTTTGCCTGTGTGTAAGTGGGGGGG-3) and ACTN3 reverse (5’-TGTCACAGTATGCAGGAGGGG-3′) were used as established by North et al. (1999) to produce a 291 base pair long DNA fragment. For detecting the ACE gene I/D gene polymorphism rs1799752, two primer combinations were used to amplify the I- and D-allele as established (Vaughan et al., 2013). For the identification of the D-allele, the primer combination (ACE1 5′-CATCCTTTTCTCCCATTTCTC-3′ and ACE3 5′-ATTTCAGAGCTGGAATAAAATT-3′) amplified a gene fragment of 83 base pairs. The I-allele was detected by the primer combination (ACE2 5′-TGGGGATTACAGGCGTGATACAG-3′ and ACE3 5′-ATTTCAGAGCTGGAATAAAATT-3′), which amplified a gene fragment of 66 base pairs.

The specificity of the method was underpinned by controls. As positive controls, the genetic material of people already genotyped for these polymorphisms was used. The negative control was a non-template control reaction with H_2_O. In addition, the reactions were always carried out in duplicate. HRM-PCR was performed using an Illumina ECO^TM^ real-time PCR system (Labgene Scientific, Châtel-St-Denis, Switzerland) with 42 cycles and described thermal settings for the PCR cycle for the respective primer pairs (North et al., 1999; Vaughan et al., 2013; Valdivieso et al., 2017a).

Gene polymorphisms were analyzed using EcoStudy software (Illumina, Labgene Scientific, Labgene Scientific, Châtel-St-Denis, Switzerland). Genotypes were identified by comparing the specific melting profiles of the samples to reference curves from the wild type, mutant and heterozygous allele, which genotypes had been identified by HRM-PCR and microsequencing. In the analysis of the ACE I/D PCR results, the amplification of the reaction mixtures with ACE I/I or ACE D/D primers was investigated. If a subject’s sample was amplified in both primer mixtures, it was identified as a heterozygous ACE-I/D genotype. However, if the sample was amplified in only one of the primers, it could be assigned to the respective homozygous genotype. If no clear result was obtained despite repetition, the amplified samples were sent to an external laboratory (Microsynth, Balgach, Switzerland) for sequencing.

### Statistics

Compliance with Hardy-Weinberg equilibrium was assessed using an MS-Excel based online calculator ^1^. Other statistical analyses were performed with statistical software SPSS 19.0 (IBM, Chicago, IL, United States). The distribution of the investigated genotypes in relation to the phenotype (power athlete, endurance athlete, non-athlete) was compared with Chi^2^ tests. Differences in muscle composition between genotypes (rs1799752, rs210477, rs1815739) and the phenotype were assessed with univariate analysis of variance (ANOVA) for single structural variables [capillary length density, capillary-to-fiber ratio, mean cross sectional area (MCSA)] and multivariate ANOVA (MANOVA) for inter-related structural variables (i.e., volume densities), respectively. Effects were localized using a *post hoc* test for the least significant difference. A *p*-value below 0.05 was considered as statistically significant. Sample size calculations were carried out with G^∗^Power for a one-way ANOVA with fixed effects (Faul et al., 2007). Linear relationships were calculated based on Pearson correlations.

## Results

### Subject Characteristics

Table 1 shows the characteristics of the endurance athletes, power athletes and non-athletes which muscle composition and genotypes were assessed. Body mass was 37%-higher in the power athletes than the endurance athletes, with the values for the non-athletes residing in between, i.e., 18% higher than in the endurance athletes.

**FIGURE 1:**
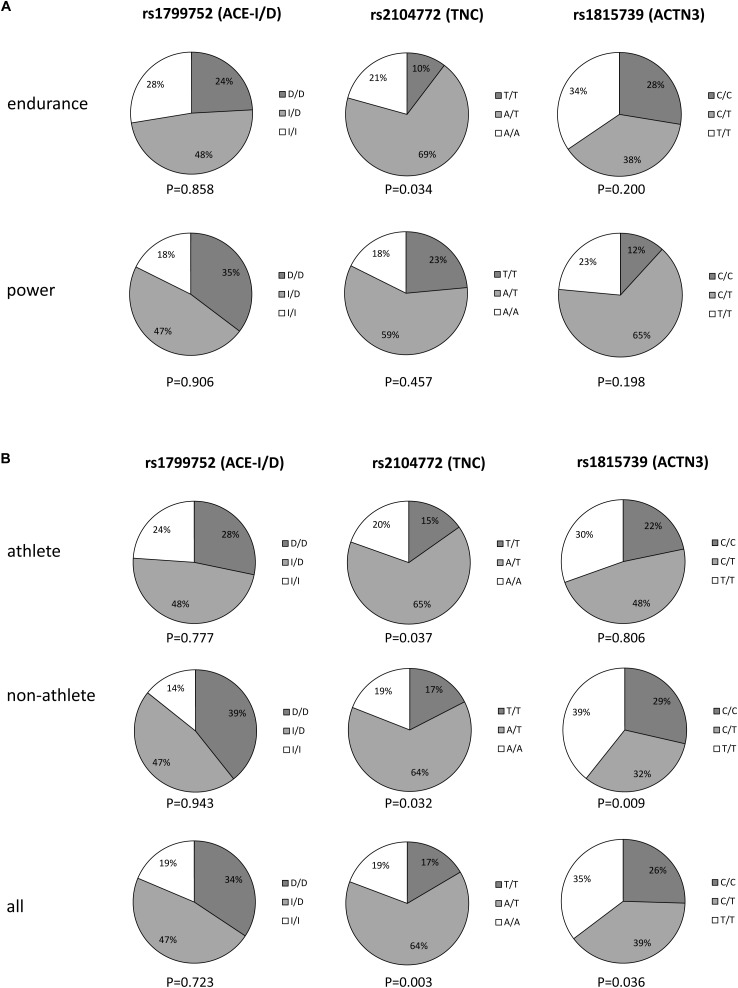
Fractions of the individual genotypes within the phenotypes. Pie chart displaying the distribution of the three genotypes in the two athlete types **(A)**, and athletes, non-athletes and all subjects combined **(B)**. *p*-values below each chart refer to the respective significance of the test for Hardy–Weinberg equilibrium.

The distribution of genotypes is shown in Figure 1. Over all phenotypes, rs1799752 (*p* = 0.723), but not rs2104772 (*p* = 0.003) and rs1815739 (*p* = 0.036), met the Hardy-Weinberg equilibrium. For rs2104772 the deviation from the equilibrium was also identified for the endurance athletes and non-athletes whereas for rs1815739 the deviation was identified only in non-athletes.

Neither genotype demonstrated a significant association with the phenotype.

### Knee Extensor Muscle Composition Between Phenotypes

**FIGURE 2:**
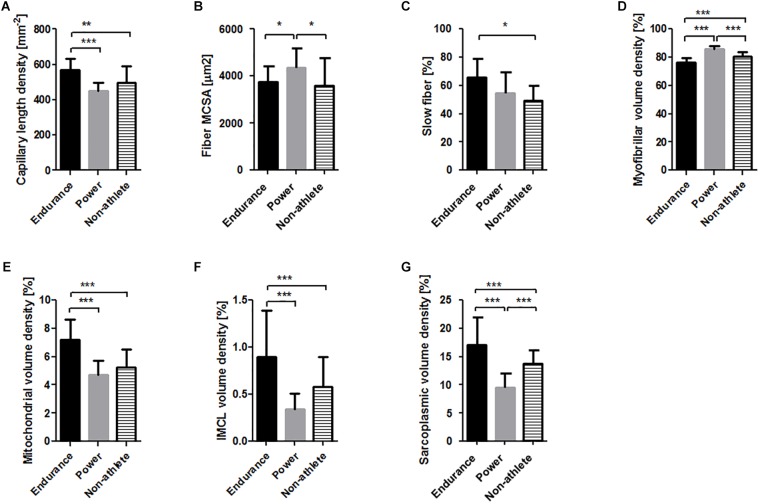
*M. vastus* lateralis composition in the three phenotypes. Bar graphs visualizing the mean and standard deviation of the **(A)** capillary length density, **(B)** muscle fiber MCSA, **(C)** slow type fiber percentage, and volume densities of myofibrils **(D)**, mitochondria **(E)**, intramyofibrillar lipid **(F)** and sarcoplasma **(G)**. Asterisk indicates significant difference. ^∗^*p* < 0.05, ^∗∗∗^*p* < 0.001.

We subsequently tested whether differences would exist between phenotypes and genotypes for muscle fibers composition and associated capillaries in *m. vastus lateralis*. Significant differences between the phenotypes, especially when only the two athlete types were considered (Table 2), were identified for all assessed muscle parameters except capillary-to-fiber (Figure 2). Capillary length density, mitochondrial volume density, intramyofibrillar lipid droplet volume density, and sarcoplasmic volume density were 26, 54, 165, and 79%, respectively higher in endurance than in power athletes. Conversely, the MCSA of muscle fibers and the myofibrillar volume density were 17, and 13%, respectively higher in power athletes. The percentage of slow type muscle fibers in the endurance athletes tended to be 11% higher than in power athletes (*p* = 0.07).

**Table 2:** Differences between power and endurance athletes for the assessed muscle parameters.

Parameter	*p*-value	*F*-value	Effect size (η^2^)
Capillary-to-fiber ratio	0.440	0.615	0.024
Capillary length density	**<0.001**	22.725	0.476
Fiber MCSA	**<0.001**	62.986	0.716
Fiber type distribution	0.069	3.671	0.149
Myofibrillar volume density	**0.035**	4.971	0.166
Mitochondrial volume density	**<0.001**	23.159	0.481
Intramyocellular lipid volume density	**0.002**	12.094	0.302
Sarcoplasmic volume density	**<0.001**	21.034	0.429


*Statistical analysis was conducted with ANOVA or MANOVA. Significant p-values are printed in bold font.*

Differences in the cellular composition of muscle fibers and capillaries of athletes respective to non-athletes pointed in opposite direction for the endurance and power athletes for the MCSA of muscle fibers, and the volume density of myofibrils and sarcoplasma, respectively. The percentage of slow type muscle fibers in the endurance athletes was 17% higher than in non-athletes.

### Genotype Effects on Muscle Specialization Are Influenced by the Phenotype

**FIGURE 3:**
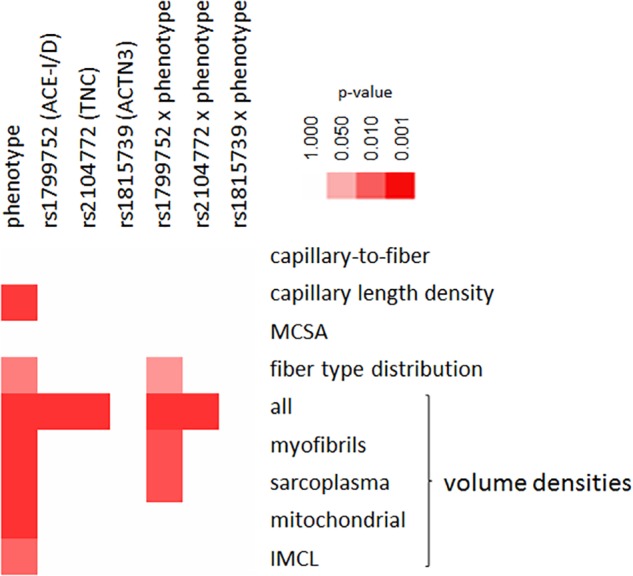
Genotype × phenotype interactions on muscle composition. Color coded heat map visualizing the statistical significance of differences between the three studied genotypes × three phenotypes for parameters of muscle composition. The color scale used to rate the level of significance is indicated to the top right.

Figure 3 visualizes the statistical significance of genotype-related differences of muscle fiber composition and associated capillaries in *m. vastus lateralis* over the three genotypes and phenotypes. rs1799752 and rs2104772 gene polymorphisms affected the multivariate volume densities. Significant associations revealed for the interaction between the phenotype and gene polymorphism rs1799752 respective to the single cellular parameters, volume densities of myofibrils and sarcoplasma, and fiber type distribution (Supplemental Table S2).

### Genotype × Phenotype Interactions in Athletes

When only considering the muscle composition of athletes, no differences between a genotype was identified (Supplemental Table S3). Power calculations exposed that the prospective replica number to identify differences in muscle composition between a genotype alone would have been in the hundreds.

When considering interaction effects between the athlete type and genotype, differences were identified for the two gene polymorphisms rs1799752 (fiber type distribution, volume densities of myofibrils and sarcoplasma, respectively) and rs2104772 (capillary length density and MCSA), but not rs1815739 (Table 3).

**Table 3:** Interactions between genotype × athlete type on muscle composition.

Comparison	Parameter	*p*-value	*F*-value	Effect size (Eta^2^)
Athlete type ×	Capillary-to-fiber ratio	0.582	0.555	0.048
rs1799752 (ACE)	**Capillary length density**	**0.023**	4.516	**0.291**
	Fiber MCSA	0.159	2.004	0.154
	Fiber type distribution	0.126	2.345	0.216
	**Myofibrillar volume density**	**0.025**	4.387	**0.285**
	Mitochondrial volume density	0.832	0.186	0.017
	Intramyocellular lipid volume density	0.234	1.545	0.114
	Sarcoplasmic volume density	0.056	3.258	0.214
Athlete type ×	Capillary-to-fiber ratio	0.879	0.130	0.012
rs2104772 (TNC)	**Capillary length density**	**0.012**	5.468	**0.332**
	Fiber **MCSA**	**0.029**	4.198	**0.276**
	Fiber type distribution	0.997	0.003	0.001
	Myofibrillar volume density	0.851	0.163	0.015
	Mitochondrial volume density	0.695	0.370	0.033
	Intramyocellular lipid volume density	0.952	0.050	0.004
	Sarcoplasmic volume density	0.938	0.065	0.005
Athlete type ×	Capillary-to-fiber ratio	0.985	0.015	0.001
rs1815739 (ACTN3)	Capillary length density	0.995	0.005	0.000
	Fiber MCSA	0.991	0.009	0.001
	Fiber type distribution	0.233	1.524	0.078
	Myofibrillar volume density	0.756	0.283	0.023
	Mitochondrial volume density	0.759	0.279	0.023
	Intramyocellular lipid volume density	0.884	0.124	0.010
	Sarcoplasmic volume density	0.550	0.013	0.049


*Statistical analysis was conducted over the two athlete types with multivariate ANOVA or univariate ANOVAs with post hoc test of least significant difference. Significant p-values and their effect sizes are printed in bold.*

**FIGURE 4:**
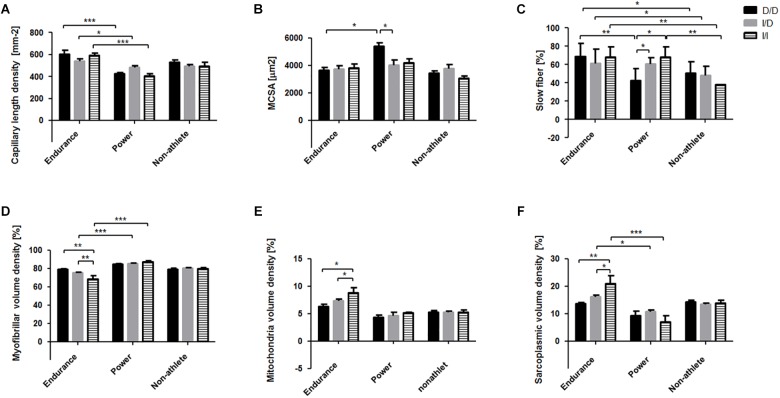
Interaction effects between the phenotype and the rs1799752 (ACE) gene polymorphism on muscle composition. Bar graph of the mean and standard deviation for the investigated muscle characteristics for the rs1799752 genotypes in the two athlete types. For comparison, the values in the non-athletes are shown as well, but without the indication of statistically significant differences. **(A)** capillary length density, **(B)** fiber mean cross sectional area (MCSA), **(C)** slow type fiber percentage, and volume densities of myofibrils **(D)**, mitochondria **(E)**, and sarcoplasmia **(F)**. ^∗^*p* < 0.05, ^∗∗^*p* < 0.01, ^∗∗∗^*p* < 0.005 for the indicated differences.

For rs1799752 the interaction effect on myofibrillar volume density could be localized to higher values in homozygous non-carriers of the ACE-I-allele (i.e., ACE-D/D genotypes) respective to ACE-I-allele carriers (i.e., heterozygous ACE-I/D and ACE-I/I genotypes) in endurance-trained athletes (Figure 4). Power-trained ACE-I-allele carriers demonstrated a higher percentage of slow type muscle fibers than those that were non-carriers of the I-allele. For capillary length density all three ACE-I/D genotypes showed higher values in those who were endurance-trained than the respective power-trained genotypes. On the *post hoc* level MCSA was higher in power-trained D/D genotypes than power-trained ACE-I-allele carriers and the respective endurance-trained D/D genotype. Sarcoplasmic volume density was higher in endurance-trained I/I genotypes than endurance-trained ACE-D-allele carriers and respective power-trained I/I genotype. As well higher volume densities of mitochondria were identified at the *post hoc* level in endurance athletes carrying the I-allele of rs1799752.

**FIGURE 5:**
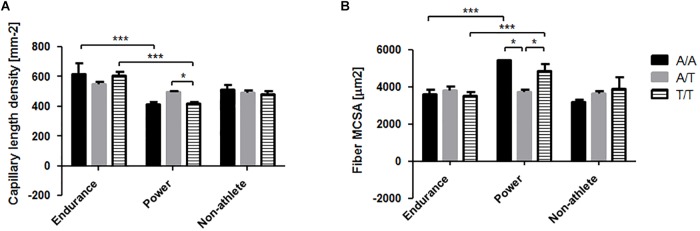
Interaction effects between the phenotype and the rs2104772 (TNC) gene polymorphism on muscle composition. Bar graph of the mean and standard deviation for the investigated muscle characteristics for the distinct rs2104772 genotypes in the two athlete types. For comparison, the values in the non-athletes are shown as well, but without the indication of statistically significant differences. **(A)** capillary length density, **(B)** fiber MCSA. ^∗^*p* < 0.05 and ^∗∗∗^*p* < 0.001 for the indicated differences.

For rs2104772 the interaction effect on capillary length density could be localized to higher values in power-trained A/T than T/T genotypes and higher values for endurance-trained A/A and T/T genotypes than the respective power-trained genotypes. For MCSA the effect could be localized to higher values in power-trained T/T compared to A/T genotypes (Figure 5). Additionally, MCSA was higher in power-trained T/T genotypes than the respective endurance-trained genotype.

A *post hoc* difference in the distribution of muscle fiber types was identified for the interaction between the rs1815739 (ACTN3) genotype and the athlete type. Thereby the percentage of slow type muscle fibers was higher in endurance-trained than power-trained T/T genotypes (*p* = 0.039) but this was not revealed for the C/C genotypes (*p* = 0.975).

## Discussion

In the past decades it has become clear that genetic predisposition may present an advantage for training-induced muscle plasticity and this may affect strategies to enhance sports-specific performance, especially in athletes (Ghosh and Mahajan, 2016). Our investigation tested a number of implicit hypotheses on associations between selected genotype × phenotype interactions and the composition of knee extensor muscle (see Supplemental Table S4). We identified differences in muscle composition that were associated with an interaction between the phenotype and gene polymorphisms rs1799752 and rs2104772. With the current sample size genotype differences for individual cellular parameters were not resolved when the phenotype was not considered (Supplemental Table S3). The results, such as those for gene polymorphism rs1799752, confirm the higher volume density for mitochondria and intramyocellular lipid in I/I vs. D/D genotypes that was previously identified in endurance-trained Swiss white Caucasian subjects (Vaughan et al., 2013). Also, phenotype-related *post hoc* differences were identified for the distribution of muscle fiber types between genotypes of polymorphism rs1799752 and rs1815739 that are in line with the reported associations with the single genotypes in untrained subjects’ muscle (see Supplemental Table S4; Zhang et al., 2003; Vincent et al., 2007). As well, we identified interaction effects between the phenotype and the gene polymorphism rs2104772, which for power athletes reproduced the expected lower muscle capillarity in T/T genotypes (Fluck et al., 2008). Interestingly, we also identified that the Hardy-Weinberg was not met for gene polymorphism rs2104772 (*p* = 0.003) in endurance athletes and non-athletes and for gene polymorphism rs1815739 in non-athletes (*p* = 0.036). The latter observations indicate an influence of mitochondria-encoded traits that are asymmetrically inherited via the maternal line on the selection and/or genotype distribution for the studied non-athletes (Dionne et al., 1991; Eynon et al., 2011). This is the first investigation addressing interaction effects between athletic phenotypes and genotypes on muscle ultrastructure in a population of highly trained subjects. Our results indicate that in the investigated population of high level athletes, alike in moderately trained subjects (Vaughan et al., 2013, 2016; Valdivieso et al., 2017b), there is a genotype related difference in the specialization of critical cellular hallmarks of muscle performance with years of training and competition.

We identified a number of differences in the composition of muscle fibers in *m. vastus lateralis* between high level endurance and power athletes and relative to untrained healthy subjects (Figure 2). In general the findings confirmed previous results on muscle specialization with types of training and are in line with the fact that certain relative variables of muscle fiber composition (such as volume densities) in either athlete population do also distinguish from the respective values in the non-athletes (see Figure 2; Hoppeler, 1986). This concerned for instance, the higher percentage of slow type muscle fibers, and higher volume densities of mitochondria, intramyocellular lipid and sarcoplasma in the muscle fibers of endurance athletes. Together with the increased capillarization, these metabolism-related adjustments reflect the cellular factors underlying the locally enhanced aerobic capacity that explain the improved fatigue resistance, and higher velocity and specific power of slow and fast type muscle fibers in elite endurance-trained subjects (Flück and Hoppeler, 2003; Zierath and Hawley, 2004; Harber and Trappe, 2008). Conversely, the elevated volume density of myofibrils and MCSA of muscle fibers in power athletes exemplifies the cellular aspects that enhance power output with (single) muscle contractions. In this regard, we note that the electron microscopically-estimated fiber MCSAs, especially those of the power athletes, were smaller than reported previously based on light microscopy (Billeter et al., 2003), reflecting methodological differences related due to tissue processing where fiber diameter is reduced by 30% vs. the original size (Carlsen et al., 1961). As well, we note that the biopsies for the studied shot-putters and weight lifters were from a knee extensor muscle where larger differences in the muscle size and fiber MCSA would probably be evident in the arm muscles compared to the non-athletes.

While there is solid evidence for the enrichment of certain genotypes of polymorphisms rs1799752 and rs1815739 in athletic cohorts compared to the general population (Yang et al., 2003; Bray et al., 2009; Ma et al., 2013; Aleksandra et al., 2016), it has been concluded lately that genotypes rs1799752 and rs1815739 may not necessarily associate at the level of statistical significance when relevant parameters for endurance athletes, such as running times in competition, are considered or when athletes are matched to fit controls (Hruskovicova et al., 2006; Ash et al., 2011; Papadimitriou et al., 2018). The athlete type-dependent differences between genotypes of polymorphisms rs1799752 and rs1815739 (and rs2104772) indicate that the enrichment of the implicated genotypes in athletic populations is related to influences on the differentiation of cellular hallmarks of mechanical and metabolic function of muscle fibers (Table 3 and Figure 4, 5). In this respect a number of the identified genotype differences are in line with expectations on the association of the three gene polymorphisms with muscle composition seen previously in untrained or moderately trained subjects (see Supplemental Table S4). For instance, for rs1815739 a higher slow type muscle fiber percentage was identified at the *post hoc* level in endurance- respective to power-trained T/T-genotypes, which was not evident in the CC-genotypes. This observation is consistent with the reported association of the T/T-rs1815739 genotype with endurance athletes and fiber type distribution in untrained subjects (Yang et al., 2003; Vincent et al., 2007). Importantly, because identified at the level of a main statistical effect, for the endurance-trained homozygous I-allele carriers of rs1799752, being associated with endurance performance (Woods et al., 2001; Flueck et al., 2010), we detected higher mitochondrial volume densities than respective D-allele carriers (Figure 4E). As well, we localized the main association of the muscle fiber type distribution with the interaction of rs1799752 and the athlete phenotype to higher percentages of slow type fiber percentage in the *m. vastii* of I-allele carrying power athletes. However, in the endurance athletes we did not identify a rs1799752 related difference in muscle fiber MCSA of *m. vastus lateralis* (Figure 4C) as previously reported for moderately endurance-trained I-allele carriers (Valdivieso et al., 2017b), and identify in contrast to our expectation (see Supplemental Table S4) a lower volume density of myofibrils in endurance athletes being homozygous for I-allele carriers compared to D-allele carriers. Consistently with the former association, a higher MCSA of muscle fibers was identified for power athletes with the ACE-D/D respective to the ACE-I/D genotype (Figure 4D). These findings suggest that the over-representation of D/D genotypes in power-type athletes (Woods et al., 2001; Flueck et al., 2010), is related to a negative association between the ACE-I allele and the concentration of myofibrils in muscle fibers in this phenotype, which reflects the main influence of the encoded ACE system on muscle fiber growth (Verbrugge et al., 2018). Meanwhile the rejection of the hypothesis, that endurance athletes carrying the I-allele would demonstrate an elevated fiber MCSA, emphasize that other factors explain the previous rs1799752-related differences in fiber MCSA of moderately endurance-trained British subject (Valdivieso et al., 2017b). It has been shown before that leg muscles (i.e., *m. vastus lateralis* and *m. gastrocnemius*) of world class shot-putters and hammer throwers are known to display a considerable range of muscle fiber compositions (Coyle et al., 1978; Terzis et al., 2010). Our observations in *m. vastus lateralis* extend this notion to suggest an association of the fiber type distribution in power athletes with gene polymorphisms in ACE and ACTN3. Collectively, our observations hint that the influence of gene polymorphism rs1799752 on the cross-sectional area (and volume) of muscle fibers and entire muscle depend on training-type modulated factors (Valdivieso et al., 2017b).

Furthermore, we identified a higher capillary length density in the *m. vastus lateralis* of A/T compared to T/T genotypes of rs2104772 for power athletes, reproducing similar genotype differences in the capillary-to-fiber ratio of moderately endurance-trained subjects (Figure 5B; Valdivieso et al., 2017a). As well, fiber MCSA was higher in T/T than A/T genotypes of rs2104772 (Figure 5B). The T/T genotype had previously been shown to demonstrate lower increases in tenascin-C protein levels in *m. vastus lateralis* with endurance training, highlighting a possible influence of tenascin-C on fiber MCSA that had been pointed out in anti-gravity muscles of tenascin-C deficient transgenic mice (Fluck et al., 2008). For rs1799752, only trends for a difference between genotypes of a given phenotype could be identified at the *post hoc* level for capillary length density in *m. vastus lateralis* (Figure 4A). For instance, in endurance athletes we observed tendinously lower values for capillary length density in I/D compared to D/D genotypes (*p* = 0.069). These observations in Swiss power athletes are consistent with our observations on similar genotype differences in capillary length density in *m. vastus lateralis* of endurance-trained healthy individuals of Swiss descent (Vaughan et al., 2013); they do however contrast findings on opposite associations in moderately endurance-trained white British subjects (Valdivieso et al., 2017b). Conversely, in power athletes capillary length density nearly tended to be lower in D/D genotypes than I/I genotypes (*p* = 0.128) of rs1799752. Overall, the athlete type modulated differences in capillary length density between genotypes of rs2104772 supports the notion of a mutual association between tenascin-C and the training-type specific adaptations in muscle capillarity. Specifically, the findings show that in power-trained A/T genotypes of rs2104772 there is a shift toward smaller fibers with better perfusion that are expected to have a metabolic advantage to carry out repeated contractions under a high metabolic flux (Figure 5). Meanwhile, the present findings do not allow us to reject the hypothesis that the ACE/angiotensin 2 system modifies vascular processes in exercised muscle, especially as this occurs in an interdependent manner with tenascin-C (reviewed in van Ginkel et al., 2016; Valdivieso et al., 2017a).

We identified that effects of the rs1799752 genotype were only resolved at the level of significance for muscle fiber type distribution, and the volume density of myofibrils and sarcoplasma in *m. vastus lateralis*, when interactions with phenotype were considered (Table 3 and Supplemental Table S2). This observation reinforces that training-type related factors importantly modify the association of the studied ACE gene polymorphism with muscle composition (Valdivieso et al., 2017b). In this respect, it is important to consider that in moderately trained subjects both the rs1799752 (and rs2104772) gene polymorphisms are associated with a different expression response of angiogenic processes as well as those of mitochondrial processes and amino acid metabolism in *m. vastus lateralis* subsequent to a single bout of endurance exercise (Vaughan et al., 2013, 2016; Valdivieso et al., 2017a,b). Collectively, the findings hint for a quantitative relevance of ACE-related genetic influences on the exercise-specific expression responses of skeletal muscle (Flueck, 2009; Vaughan et al., 2013; Mathes et al., 2015) which remains to be addressed.

Differences in the organellar composition of the knee extensor muscle, as they relate to the metabolic and mechanical properties of skeletal muscle (Weibel, 1996; Weibel and Hoppeler, 2005), may reflect to a considerable degree the variability of muscular aspects of physical performance (Reid and Fielding, 2012). In this respect it is worth to consider that correlations existed between genotype-associated cellular and subcellular parameters of knee extensor muscle and physiological parameters of aerobic performance over the entire population of endurance athletes and non-athletes (Supplemental Figure S2). This concerned positive linear relationships between VO_2_max and aerobic peak power output, with capillary-to-fiber ratio (*r* = 0.56 and 0.53) and mitochondrial volume density (*r* = 0.76 and 0.47), and conversely negative linear relationships of the former two physiological parameters with the volume density of myofibrils (*r* = -0.74 and -0.40), all of which met p-values of below 0.02. Also, the percentage of slow type muscle fibers (*r* = 0.53, *p* = 0.001) and sarcoplasmic volume density (*r* = 0.64, *p* < 0.001) were linearly related to VO_2_max, supporting the contribution of muscle composition to aerobic capacity and performance (Tihanyi et al., 1982; di Prampero, 2003; Pollock et al., 2018; van der Zwaard et al., 2018). In this respect it is worth to consider that certain differences in muscle composition between genotypes of rs1799752 and rs2104772 within a given athlete phenotype were considerable. For instance the volume density of mitochondria was approximately 50% higher in ACE I-allele carriers compared to ACE-D/D genotypes (Figure 4E). As well in ACE I-allele carriers, 50% higher values were identified for the volume density of sarcoplasma (Figure 4F), the functional relevance of which is not understood. Similarly, capillary length density, reflecting the capacity for substrate supply to working muscle (Vock et al., 1996), was nearly 25% higher in A/T genotypes of rs2104772 when MSCA demonstrated the inverse trend (Figure 5). These differences indicate that an increased capacity for capillary perfusion and mitochondrial metabolism in knee extensor muscle contributes to the reportedly elevated aerobic capacity for athletic I-allele carriers of polymorphism rs1799752 and suggests a similarly improved capacity for fueling substrate import in A/T carriers of rs2104772. Previous investigations found associations between the ACE and ACTN3 polymorphism and physical performance (Woods et al., 2001; Yang et al., 2003; Kikuchi et al., 2014) while others did not report an association between the ACE gene I/D polymorphism and the multisystemic variable VO_2_max in endurance athletes *per se* (Rankinen et al., 2000). In this respect it has been shown that the incidence of the ACE-I/I genotype is increased in successful marathon runners and inline skaters (Hruskovicova et al., 2006). It remains to be elaborated to which degree the in here identified genotype x phenotype interaction effects on knee extensor muscle composition, such as the elevated volume density of mitochondria in the highly endurance-trained ACE-I/I genotypes (Figure 4), stand for differences in local strategies between genotypes to enhance muscle performance of athletes, and excel in competition.

All metadata on skeletal muscle composition were collected using standardized methods (Supplemental Table S1), legitimizing the direct comparison of the results from different data sets. In order to ensure that the study results are not biased, the selected athletes are only elite athletes, who rank among the national elite or even among the world leaders in their discipline. Our study however was subject to limitations which reduce the statistical and interpretative power of our results for the competitive athlete. For instance, because it is difficult to obtain biopsies from elite athletes, the study population of 46 elite athletes for a gene association study is rather small. The consequently lowered power explains why our investigation could not confirm (at the level of statistical significance) the reported associations for the single gene polymorphisms rs1799752 and rs1815739 in endurance- respective to power-athletes that have been shown in studies with large cohorts (Woods et al., 2000; Vincent et al., 2007; Bray et al., 2009; Kikuchi et al., 2014; Aleksandra et al., 2016). For fiber type distribution we could extend previous findings on the association of gene polymorphisms rs1799752 and rs1815739 and slow fiber percentage to elite athletes (Yang et al., 2003; Zhang et al., 2003; Vincent et al., 2007; Verbrugge et al., 2018). To counteract limitations due to sample size, and in line with others (Aleksandra et al., 2016) we pooled data from different type of endurance athletes (i.e., cyclist and runner) despite the possibly different degree of ultra-structural specialization in knee extensor muscle (Vaughan et al., 2013). Secondly, we do not have a complete set of physiological or performance records, especially as these were not recorded at the time of biopsy collection, and because the anonymous data assessment did not allow completing missing data. Thus, the relevance of volumetric differences in muscle organelles in the studied knee extensor muscle in setting variability of athletic performance cannot be fully appreciated or addressed. The identified differences in muscle composition between endurance and power-type athletes, and the interaction between genotype differences, are to a good degree in line with previous studies in non-athletes (Valdivieso et al., 2017a,b), and support that the pooling of *vastii* between different sports disciplines with similar “power” characteristics did not essentially interfere with the resolution of biologically relevant genetic influences. Nevertheless. the identified statistically significant interaction effects between the ACE and *post hoc* differences support the notion of a heritable influence on muscle composition depending on the athletic phenotype. The underlying mechanism, i.e., whether this reflects an adaptive response to the years of training and competition, and the extent to which the resulting genetic differences in muscle composition are of functional relevance to produce differences in muscle’s capacity to produce power over short or long duration, remains to be elaborated. Possibly this also comprises the investigation of further gene polymorphisms to apprehend the breadth of the possible genetic influence on training-specific adaptations in muscle composition.

## Conclusion

Polymorphisms in the genes for angiotensin-converting enzyme and tenascin-C are confirmed to be associated with the specialization of (sub)cellular aspects of metabolic and mechanical muscle functioning and show that this interacts with the athlete phenotype. Reported associations between the studied gene polymorphisms and differences in the acute effects of physical activity on muscle gene expression and muscle adaptation hint that the identified genotype × athlete phenotype interactions are due to the impact of physiological cues during years of training and competition. Our results may contribute to the development of a future personalized approach to physical training which takes the trainability of metabolic and contractile traits of muscle performance into account. Currently, however, the functional relevance of the identified genotype influence between endurance and power athletes for muscle performance remain to be further analyzed.

## Ethics Statement

The study was conducted in accordance with the Helsinki declaration for research on humans. The gathering of physiological and cell biological meta-data was conducted with permission of the Ethics committee of the canton of Berne. Genotyping was carried out and analyzed anonymously under application of the non-competence rule of the Human Research Act after clarification by the ethics committee of the canton of Zurich.

## Author Contributions

MF designed the study. MVF acquired the funding. MVF, PV, SK, and MK performed the experiments. MK and PV analyzed the experiments. MVF, MK, SK, and PV analyzed the data. DF interpreted the results. MVF, MK, and DF drafted the manuscript. MF, MVF, and PV revised the manuscript. MF, MK, DF, SK, MVF, and PV endorsed the manuscript.

## Conflict of Interest Statement

The authors declare that the research was conducted in the absence of any commercial or financial relationships that could be construed as a potential conflict of interest.

## Footnotes

1 www.dr-petrek.eu/documents/HWE.xls

## Abbreviations

ACE: angiotensin converting enzyme
ACTN3: actinin-3
HRM-PCR: high resolution melt analysis polymerase chain reaction
MCSA: mean cross sectional area
TNC: tenascin-C
VO_2_max: maximal oxygen uptake
