# Supplementary material for: Cellular Aspects of Muscle Specialization Demonstrate Genotype – Phenotype Interaction Effects in Athletes
Source: Front Physiol. 2019 May 8;10:526. doi: 10.3389/fphys.2019.00526 (PMC6518954; doi:10.3389/fphys.2019.00526)
Supplement: Supplementary file 1 [file Table_1.docx]

***Supplemental table S1:*** *Relationships of morphometric measurements to previous reports.* The list attributes the analyzed data on muscle composition (indicated with an ‘X’ in the different athlete phenotypes) to previous publications.

| **phenotype** | *capillary length density* | *capillary-to-fiber ratio* | *fiber MCSA* | *myofibrillar volume density* | *sarcoplasmic volume density* | *mitochondrial volume density* | *intramyocellular lipid volume density* | *fiber type distribution* | ***Reference*** |
| --- | --- | --- | --- | --- | --- | --- | --- | --- | --- |
| **Endurance athlete** | X | X | X | X | X | X | X | X | ([37](#_ENREF_37), [44](#_ENREF_44), [51-53](#_ENREF_51)) |
|  |  |  |  |  |  |  |  |  |  |
| **Power athlete** | X | X | X | X | X | X | X | X | ([45](#_ENREF_45), [54](#_ENREF_54)) |
|  |  |  |  |  |  |  |  |  |  |
| **Non-athlete** | X | X | X | X | X | X | X | X | ([47](#_ENREF_47), [55-57](#_ENREF_55)) |
