# Supplementary material for: Cellular Aspects of Muscle Specialization Demonstrate Genotype – Phenotype Interaction Effects in Athletes
Source: Front Physiol. 2019 May 8;10:526. doi: 10.3389/fphys.2019.00526 (PMC6518954; doi:10.3389/fphys.2019.00526)
Supplement: Supplementary file 2 [file Table_2.docx]

***Supplemental table S2:*** *Interactions between the three genotype x phenotype on muscle composition.* Statistical analysis was conducted over all studied subjects with multivariate or univariate ANOVAs with post hoc test of least significant difference. Significant p-values and their effect sizes are printed in bold.

| **Comparison** | **Parameter** | ***p*-value** | ***F*-value** | **Effect size (Eta^2^)** |
| --- | --- | --- | --- | --- |
| rs1799752 | Capillary-to-fiber ratio | 0.5197 | 0.6626 | 0.0244 |
| (ACE) | Capillary length density | 0.8097 | 0.2117 | 0.0058 |
|  | Fiber MCSA | 0.5159 | 0.6678 | 0.0180 |
|  | Fiber type distribution | 0.6771 | 0.3940 | 0.3800 |
|  | **Volume densities** | **0.0004** | **5.8540** | **0.2429** |
|  | Myofibrillar volume density | 0.2821 | 1.2872 | 0.0332 |
|  | Mitochondrial volume density | 0.1372 | 2.0401 | 0.0516 |
|  | Intramyocellular lipid volume density | 0.0982 | 2.3938 | 0.0600 |
|  | Sarcoplasmic volume density | 0.4087 | 0.9054 | 0.0236 |
|  |  |  |  |  |
| rs2104772 | Capillary-to-fiber ratio | 0.8190 | 0.2004 | 0.0075 |
| (TNC) | Capillary length density | 0.8198 | 0.1993 | 0.0054 |
|  | Fiber MCSA | 0.4437 | 0.8217 | 0.0220 |
|  | Fiber type distribution | 0.1835 | 1.7677 | 0.0794 |
|  | **Volume density** | **<0.0001** | **8.6300** | **0.3210** |
|  | Myofibrillar volume density | 0.7985 | 0.2257 | 0.0060 |
|  | Mitochondrial volume density | 0.7257 | 0.3220 | 0.0085 |
|  | Intramyocellular lipid volume density | 0.1000 | 2.3749 | 0.0596 |
|  | Sarcoplasmic volume density | 0.7366 | 0.3070 | 0.0081 |
|  |  |  |  |  |
| rs1815739 | Capillary-to-fiber ratio | 0.9878 | 0.0123 | 0.0005 |
| (ACTN3) | Capillary length density | 0.9513 | 0.0500 | 0.0014 |
|  | Fiber MCSA | 0.8278 | 0.1895 | 0.0052 |
|  | Fiber type distribution | 0.8475 | 0.1662 | 0.0087 |
|  | Volume density | 0.1725 | 1.6440 | 0.0826 |
|  | Myofibrillar volume density | 0.5922 | 0.5276 | 0.0139 |
|  | Mitochondrial volume density | 0.1497 | 1.9482 | 0.0494 |
|  | Intramyocellular lipid volume density | 0.5792 | 0.5502 | 0.0145 |
|  | Sarcoplasmic volume density | 0.7256 | 0.3222 | 0.0085 |
|  |  |  |  |  |
| phenotype * | Capillary-to-fiber ratio | 0.8957 | 0.2705 | 0.0200 |
| rs1799752 | Capillary length density | 0.3571 | 1.1128 | 0.0575 |
| (ACE) | Fiber MCSA | 0.3939 | 1.0375 | 0.0538 |
|  | **Fiber type distribution** | **0.0292** | **3.0264** | **0.2416** |
|  | **Volume density** | **<0.0001** | **8.1510** | **0.3030** |
|  | **Myofibrillar volume density** | **0.0027** | **4.4672** | **0.1924** |
|  | Mitochondrial volume density | 0.2421 | 1.4004 | 0.0695 |
|  | Intramyocellular lipid volume density | 0.0511 | 2.4791 | 0.1168 |
|  | **Sarcoplasmic volume density** | **0.0027** | **4.4681** | **0.1924** |
|  |  |  |  |  |
| phenotype * | Capillary-to-fibre ratio | 0.9769 | 0.1145 | 0.0086 |
| rs2104772 | Capillary length density | 0.2924 | 1.2629 | 0.0647 |
| (TNC) | Fiber MCSA | 0.3518 | 1.1243 | 0.0580 |
|  | Fiber type distribution | 0.8953 | 0.2705 | 0.0257 |
|  | **Volume density** | **<0.0001** | **13.6530** | **0.4214** |
|  | Myofibrillar volume density | 0.7688 | 0.4545 | 0.0237 |
|  | Mitochondrial volume density | 0.3471 | 1.1339 | 0.0570 |
|  | Intramyocellular lipid volume density | 0.7426 | 0.4906 | 0.0255 |
|  | Sarcoplasmic volume density | 0.8206 | 0.3823 | 0.0200 |
|  |  |  |  |  |
| phenotype * | Capillary-to-fiber ratio | 0.9026 | 0.2594 | 0.0192 |
| rs1815739 | Capillary length density | 0.2765 | 1.3042 | 0.0667 |
| (ACTN3) | Fiber MCSA | 0.9887 | 0.0783 | 0.0043 |
|  | Fiber type distribution | 0.2747 | 1.3434 | 0.0959 |
|  | Volume density | 0.0522 | 2.4650 | 0.1162 |
|  | Myofibrillar volume density | 0.8802 | 0.2953 | 0.0155 |
|  | Mitochondrial volume density | 0.8369 | 0.3591 | 0.0188 |
|  | Intramyocellular lipid volume density | 0.2286 | 1.4418 | 0.0714 |
|  | Sarcoplasmic volume density | 0.6007 | 0.6907 | 0.0355 |
