# Supplementary material for: Cellular Aspects of Muscle Specialization Demonstrate Genotype – Phenotype Interaction Effects in Athletes
Source: Front Physiol. 2019 May 8;10:526. doi: 10.3389/fphys.2019.00526 (PMC6518954; doi:10.3389/fphys.2019.00526)
Supplement: Supplementary file 3 [file Table_3.docx]

***Supplemental table S3:*** *Genotype-related differences in assessed muscle parameters over power and endurance athletes combined.* Statistical analysis of p-values, F-values, and effect size, was conducted with multivariate ANOVA or univariate ANOVAs with post hoc test of least significant difference. Sample size refers to the prospective number of biological replicas to achieve statistical significance.

| **factor** | **parameter** | ***p*-value** | ***F*-value** | **effect size (η^2^)** | **sample size** |
| --- | --- | --- | --- | --- | --- |
| rs1799752 | Capillary-to-fiber ratio | 0.875 | 0.134 | 0.012 | 1275 |
| (ACE) | Capillary length density | 0.384 | 1.000 | 0.083 | 174 |
|  | Fiber MCSA | 0.672 | 0.405 | 0.036 | 417 |
|  | Fiber type distribution | 0.241 | 1.551 | 0.154 | 90 |
|  | Myofibrillar volume density | 0.707 | 0.352 | 0.031 | 486 |
|  | Mitochondrial volume density | 0.304 | 1.256 | 0.103 | 138 |
|  | Intramyocellular lipid volume density | 0.075 | 2.849 | 0.174 | 78 |
|  | Sarcoplasmic volume density | 0.141 | 2.108 | 0.135 | 105 |
|  |  |  |  |  |  |
| rs2104772 | Capillary-to-fiber ratio | 0.764 | 0.272 | 0.024 | 633 |
| (TNC) | Capillary length density | 0.886 | 0.122 | 0.011 | 1392 |
|  | Fiber MCSA | 0.165 | 1.954 | 0.151 | 90 |
|  | Fiber type distribution | 0.437 | 0.870 | 0.093 | 156 |
|  | Myofibrillar volume density | 0.922 | 0.081 | 0.007 | 2196 |
|  | Mitochondrial volume density | 0.770 | 0.264 | 0.023 | 660 |
|  | Intramyocellular lipid volume density | 0.069 | 2.951 | 0.179 | 75 |
|  | Sarcoplasmic volume density | 0.236 | 1.523 | 0.101 | 141 |
| rs1815739 | Capillary-to-fiber ratio | 0.627 | 0.478 | 0.042 | 357 |
| (ACTN3) | Capillary length density | 0.881 | 0.127 | 0.011 | 1382 |
|  | Fiber MCSA | 0.232 | 1.563 | 0.124 | 114 |
|  | Fiber type distribution | 0.496 | 0.729 | 0.075 | 195 |
|  | Myofibrillar volume density | 0.464 | 0.795 | 0.067 | 219 |
|  | Mitochondrial volume density | 0.320 | 1.199 | 0.098 | 147 |
|  | Intramyocellular lipid volume density | 0.164 | 1.937 | 0.125 | 114 |
|  | Sarcoplasmic volume density | 0.669 | 0.407 | 0.029 | 522 |
