# Supplementary material for: Cellular Aspects of Muscle Specialization Demonstrate Genotype – Phenotype Interaction Effects in Athletes
Source: Front Physiol. 2019 May 8;10:526. doi: 10.3389/fphys.2019.00526 (PMC6518954; doi:10.3389/fphys.2019.00526)
Supplement: Supplementary file 4 [file Table_4.docx]

***Supplemental table S4:*** List of the implicit research hypotheses, the reference for its foundation and the outcome of our investigation regarding its validity.

***hypothesis***

***polymorphism gene cellular parameter statement confirmed withheld rejected reference***

rs1799752 ACE fiber type distribution genotype x phenotype effects yes yes no ([30](#_ENREF_30))

rs1799752 ACE MCSA genotype x phenotype effects no yes no ([30](#_ENREF_30))

rs1799752 ACE MCSA ACE I-allele carriers > ACE-DD no yes no ([20](#_ENREF_20), [30](#_ENREF_30))

for endurance athletes

rs1799752 ACE capillarisation ACE I-allele carriers > ACE-DD no yes no ([30](#_ENREF_30))

for endurance athletes

rs1799752 ACE slow fiber type % ACE I-allele carriers > ACE-DD yes yes no ([32](#_ENREF_32), [33](#_ENREF_33))

rs1799752 ACE mitochondrial volume ACE I-allele carriers > ACE-DD yes yes no ([21](#_ENREF_21))

rs2104772 TNC capillarisation T/T < A-allele carriers no yes no ([22](#_ENREF_22), [31](#_ENREF_31))

for endurance athletes

rs1815739 ACTN3 slow fiber type % T/T > C-allele carriers no yes no ([17](#_ENREF_17), [34](#_ENREF_34))

especially in endurance athletes
