# Supplementary figures and images for: Cellular Aspects of Muscle Specialization Demonstrate Genotype – Phenotype Interaction Effects in Athletes
Source: Front Physiol. 2019 May 8;10:526. doi: 10.3389/fphys.2019.00526 (PMC6518954; doi:10.3389/fphys.2019.00526)

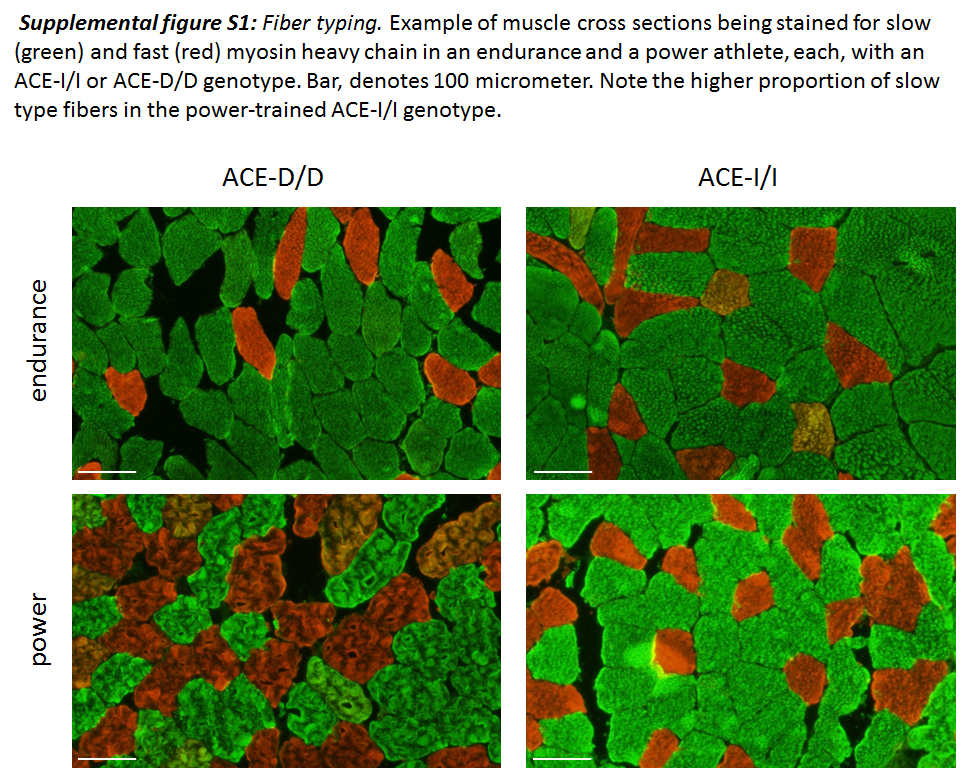

Supplement: Supplementary file 5 [file Image_1.TIF]

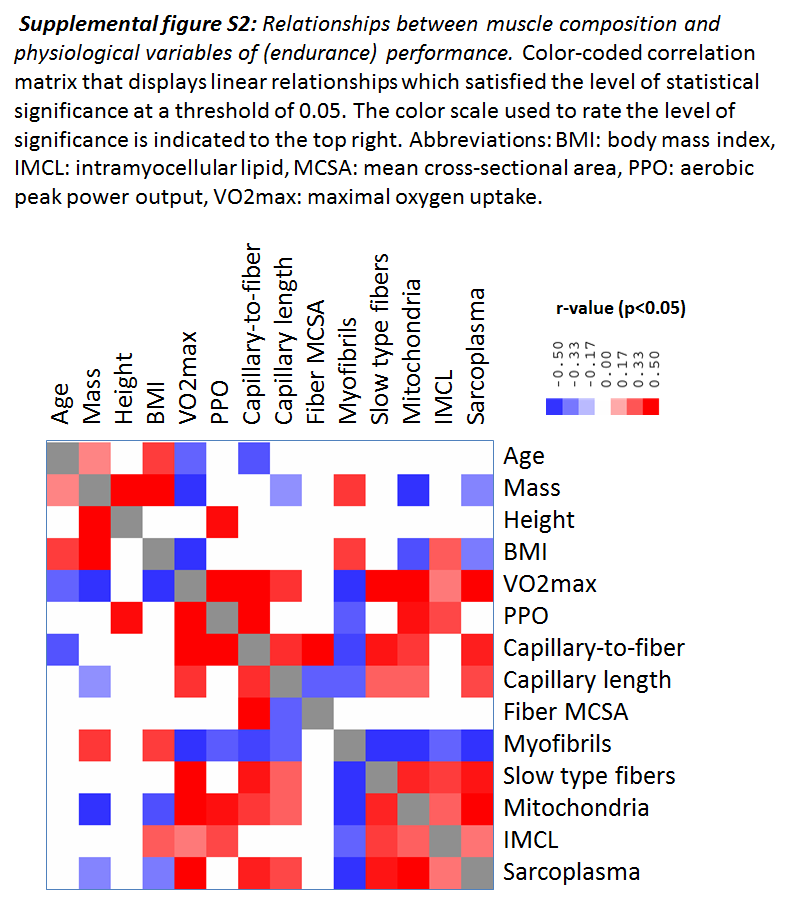

Supplement: Supplementary file 6 [file Image_2.TIF]
